# Quantification of Unmethylated Insulin DNA Using Methylation Sensitive Restriction Enzyme Digital Polymerase Chain Reaction

**DOI:** 10.3389/ti.2022.10167

**Published:** 2022-04-07

**Authors:** Fenna E. M. van de Leemkolk, Rogier J. Nell, Mieke Versluis, Eelco J. P. de Koning, Volkert A. L. Huurman, Ian P. J. Alwayn, Rutger J. Ploeg, Pieter A. van der Velden, Marten A. Engelse

**Affiliations:** ^1^ LUMC Transplant Center, Leiden University Medical Center, Leiden, Netherlands; ^2^ Department of Surgery, Leiden University Medical Center, Leiden, Netherlands; ^3^ Department of Ophthalmology, Leiden University Medical Center, Leiden, Netherlands; ^4^ Department of Internal Medicine, Leiden University Medical Center, Leiden, Netherlands; ^5^ Nuffield Department of Surgical Sciences, University of Oxford, Oxford, United Kingdom

**Keywords:** transplantation, biomarker, unmethylated insulin DNA, methylation sensitive restriction enzyme, digital PCR, β-cell, islets

## Abstract

Assessment of specific β-cell death can be used to determine the quality and viability of pancreatic islets prior to transplantation and hence predict the suitability of the pancreas for isolation. Recently, several groups have demonstrated that unmethylated insulin (*INS*)-DNA is correlated to β-cell death in type 1 diabetes patients and during clinical islet isolation and subsequent transplantation. Here, we present a step-by-step protocol of our novel developed method for quantification of the relative amount of unmethylated *INS*-DNA using methylation sensitive restriction enzyme digital polymerase chain reaction This method provides a novel and sensitive way to quantify the relative amount of β-cell derived unmethylated *INS*-DNA in cellular lysate. We therefore suggest that this technique can be of value to reliably determine the purity of an islet preparation and may also serve as a measure of the quality of islets prior to transplantation measuring unmethylated *INS*-DNA as a reflection of the relative amount of lysed β-cells.

## Introduction

β-cell replacement therapy has been established as a therapy for patients with complex Type 1 Diabetes (T1D) not amenable to optimal conventional diabetes management (1). One example of β-cell replacement therapy is the transplantation of deceased donor derived pancreatic islets that has proven its long-term efficacy during the past 20 years (2, 3). In order to aim for optimal post-transplant outcomes, the use of high-quality pancreatic islets is essential. Reliable assays are needed to assess the quality and viability of islets prior to transplantation. Soluble β-cell specific biomarkers may serve as a relevant diagnostic target to determine the quality and viability of islets at an early stage as they can be used to assess the amount of β-cell loss during islet isolation and subsequent transplantation.

Recently, several groups have reported unmethylated Insulin (*INS*)-DNA as a specific β-cell death marker during the early development of T1D. During the progression of the disease, autoimmune destruction of β-cells occurs and unmethylated *INS*-DNA is released in the bloodstream that can be identified (4-11). As the concentration of this marker is extremely low, digital polymerase chain reaction (PCR) is often used to detect the amount of β-cell death in a quantitative manner. Recent studies using digital PCR to analyze unmethylated *INS*-DNA were based on a sodium-bisulfite conversion method that chemically converts unmethylated cytosine into uracil (6, 8-10, 12). However, this method comprises an insurmountable problem as regards heterogeneity since it depends on the completeness of the chemical conversion. Overshooting or incomplete bisulfite conversion can lead to reduced sensitivity and may hamper quantitative and qualitative interpretation (13).

To avoid bisulfite conversion whilst still allowing the possibility to specifically quantify the methylation fraction of a specific allele, we recently published a methylation sensitive restriction enzyme (MSRE) digital PCR assay (14). MSREs are used to differentiate between methylated and unmethylated alleles and in combination with digital PCR it provides the opportunity to determine specific allele quantification.

Based on this methodology we now describe here the step-by-step approach how to quantify the unmethylated *INS-*DNA fraction using a MSRE and digital PCR assay. In this proof-of-concept study, we aim to demonstrate that this novel assay can be used as a helpful method to determine the purity of an islet preparation by measuring the amount of β-cells specific genomic DNA in an islet suspension. The subsequent step to then test this particular assay as a clinically quality marker of islets prior to transplantation by measuring the relative amount of lysed β-cells was beyond the scope of this proof-of-concept study. .

## Method

### Sample Collection and DNA Isolation

Human insulinoma EndoC-βH1 cells (Univercell-Biosolutions (15), Toulouse, France) and human monocytic THP-1 cells (Invivogen, Toulouse, France) were used as a positive and negative control, respectively. Isolated human pancreatic islets with different purities were obtained from seven individual pancreases (Leiden University Medical Center, Netherlands). Human donor pancreases were used that were declined for clinical purposes according to national criteria. Written informed consent for research of pancreatic tissue from donors was present, according to local guidelines of the medical ethical committee (Leiden University Medical Center, Netherlands) and of the Dutch Transplantation Foundation as the competent authority for organ donation in Netherlands. Regarding the culture of the EndoC-βH1 and THP-1 cells and isolation and maintenance of human islets, please find further details in the Supplemental document.

1) Stored pellets of 2.5 × 10^^^6 EndoC-βH1 cells, 2.5 × 10^^^6 THP-1 cells and 10 µL tissue of different purities from human islets were resuspended with phosphate buffer up to a final volume of 200 µL.

From these samples genomic DNA was extracted using a QIAamp DNA Mini Kit (Qiagen, Venlo, Netherlands) according to the manufacturer’s instructions.

2) DNA concentrations were measured using NanoDrop TM 1000 Spectrophotometer (Thermo Fisher Scientific, Landsmeer, Netherlands).

### Treatment With Methylation Sensitive Restriction Enzyme

**FIGURE 1:**
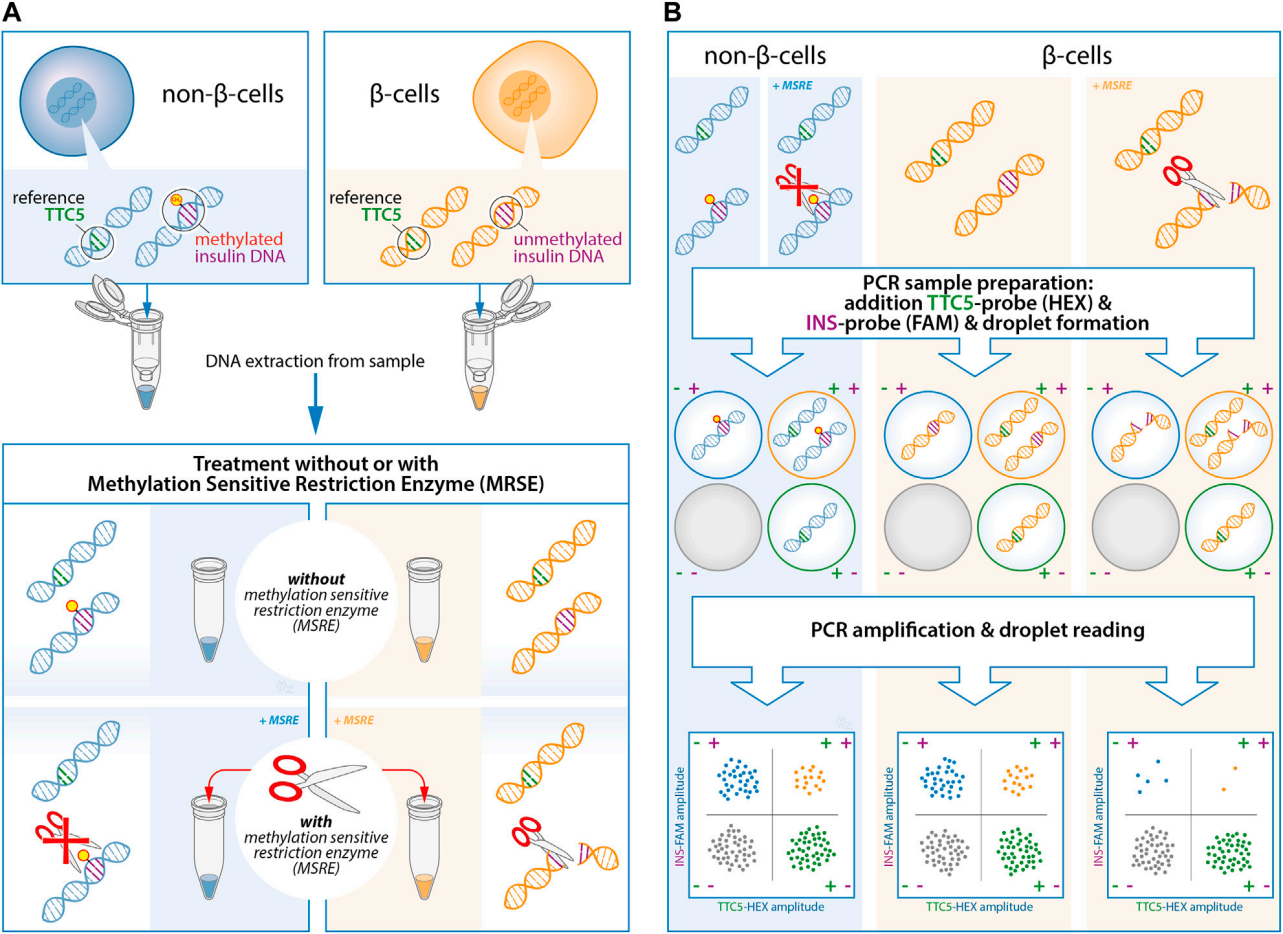
Experimental design of the quantification of unmethylated insulin (*INS*)-DNA using methylation sensitive restriction enzyme (MSRE) digital droplet polymerase chain reaction (ddPCR). **(A)** DNA is isolated from the samples and subsequently split in two and treated with or without the MSRE. The MSRE cleaves the unmethylated *INS*-DNA and leaves the methylated *INS*-DNA intact. **(B)** The DNA sample is partitioned into thousands of droplets followed by PCR amplification with FAM-labelled hydrolysis probes directed to the *INS target DNA* and probes directed to a reference gene (HEX-labelled). Droplet reading takes place after amplification. Droplets that are positive or negative for the *INS target DNA* and/or reference gene are counted to calculate the fraction of unmethylated *INS*-DNA in the sample. Abbreviations: ddPCR, Digital Droplet polymerase Chain Reaction; INS, Insulin; MSRE, Methylation Sensitive Restriction Enzyme; PCR, polymerase Chain Reaction.

The restriction enzyme, HpaII (Thermo Fisher Scientific), was used according to manufacturer’s instructions. The restriction enzyme was added for the *INS target DNA* (Figure 1A) as it cleaves the unmethylated *INS*-DNA and leaves the methylated *INS*-DNA intact. Each sample was either left untreated or treated with HpaII.

1) Take two separate units of 100 ng genomic DNA from each sample and add each of these units to a separate PCR tube (8-strip PCR tubes). Mark the first strip as “with MSRE” and the second strip as “without MSRE”. Include at least one sample in each strip containing only nuclease-free H_2_O (negative control).
2) Add 2 units/reaction of HpaII, 1.0 µL CutSmart Buffer (Bioké, Leiden, Netherlands), and nuclease-free H_2_O up to a total volume of 10 µL to the strip marked as “with MSRE".
3) Add 1.0 µL CutSmart Buffer (Bioké, Leiden, Netherlands), and nuclease-free H_2_O up to a total volume of 10 µL to the strip marked as “without MSRE".
4) Incubate both strips at 37°C for 1 hour.

### Duplex Analysis Using Digital PCR

Primers and FAM-labelled hydrolysis probes (both Sigma-Aldrich) were designed to be 1) gene specific, 2) to contain an MSRE specific CpG site and 3) to possess optimal melting temperature (±55°C) based on the region identified previously (Supplementary Figure S1) (11, 16). Probes directed to the *INS target DNA* were labelled with FAM (Supplementary Table S1). The probe directed to the reference TTC5 (tetratricopeptide repeat domain 5) gene was labelled with HEX (BioRad, Veenendaal, Netherlands).

1) To prepare the PCR mastermix, add 11 µL per reaction of Droplet PCR Supermix™ (No dUTP) (BioRad) (e.g., 110 µL per 10 samples), 0.5 µL per reaction 36uM forward *INS* primer (e.g., 5 µL per 10 samples), 0.5 µL per reaction 36uM *INS* reverse primer (e.g., 5 µL per 10 samples), 0.5 µL per reaction 10uM *INS* FAM probe (e.g., 5 µL per 10 samples), 1 µL per reaction 20x TTC5 HEX assay (e.g., 10 µL per 10 samples) and 6.5 µL per reaction nuclease-free H_2_O (e.g., 65 µL per 10 samples).
2) In order to set up a PCR reaction in a 96-well plate, first, add 20 µL mastermix to each well. Add 2 µL of cleaved unmethylated *INS*-DNA (from the “with MSRE” PCR-strip) or uncleaved unmethylated *INS*-DNA (from the “without MSRE” PCR-strip) to each appropriate well. Mix wells by pipetting up-and-down several times.

All eight wells in a column must contain cleaved unmethylated *INS*-DNA (from the “with MSRE” PCR-strip) or uncleaved unmethylated *INS*-DNA (from the “without MSRE” PCR-strip).

3) Seal the 96-well PCR plate with foil and centrifuge shortly to remove liquid from the sides of the wells.
4) Digital PCR is performed using the digital droplet PCR (ddPCR) method described below (Figure 1B).
  4.1) Use the Automated Droplet Generator (BioRad) to generate droplets according to manufacturer’s instructions.
  4.2) In order to prevent evaporation of the newly formed droplets, the droplets should be collected in a second 96-well PCR plate placed into a properly frozen cooling block.
  4.3) When finished, remove the 96-well PCR plate including the newly formed droplets and use a Plate Sealer (BioRad) in order to cover the 96-well PCR plate with a heat-sealed foil.

NOTE: Careful handling is strongly advised as the newly formed droplets are fragile in this stage.

5) Perform a PCR reaction in a T100 Thermal Cycler (BioRad) using the following protocol:
  • 10 min of activation at 95°C
  • 30s at 94°C denaturation and 60s at 60°C for 40 cycles
  • 10 min inactivation at 98°C
  • Cooling at 12°C until droplet reading
6) Analyze the DNA content of the droplets using the QuantaSoft™ software with the QX200 Droplet Reader (BioRad) according to manufacturer’s protocol.
7) Calculate for each sample the unmethylated *INS-*DNA fraction as follows:
  • Unmethylation fraction = $1-\frac{\frac{\left[INS\right]}{\begin{array}{c} \left[TTC5\right] \end{array}}\;with\;MSRE}{\frac{\begin{array}{c} \left[INS\right] \end{array}}{\left[TTC5\right]}\;without\;MSRE\;}*100\%$

## Results

With attention to previous studies (11, 16) on target areas of DNA methylation in the human *INS* gene, we designed a methylation sensitive restriction enzyme (MSRE) duplex digital PCR assay to determine the relative amount of unmethylated *INS*-DNA fraction in our DNA samples of interest.

**FIGURE 2:**
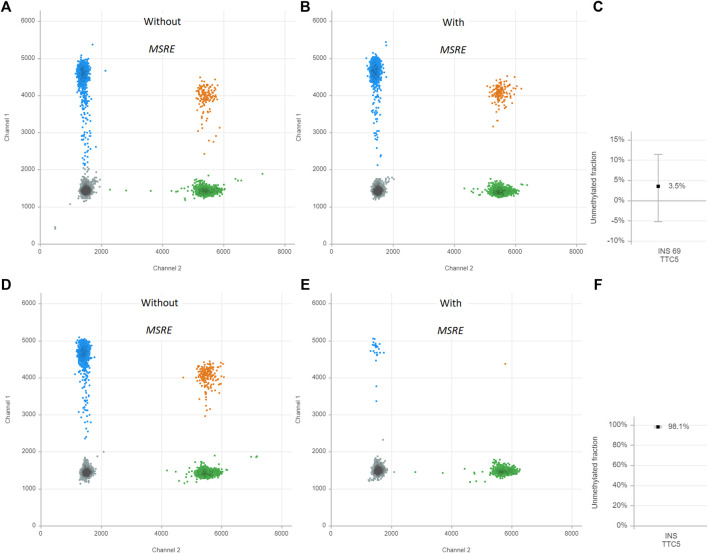
Unmethylated *INS*-DNA fraction in EndoC-βH-1 cells **(A–C)** and THP-1 cells **(D–F)** as positive and negative control samples, respectively. The two-dimensional plots from the digital droplet polymerase chain reaction (ddPCR) demonstrate four different clusters each representing different DNA containing droplets: Gray cluster: FAM- HEX-, containing no *INS target DNA* and no reference copies. Green cluster: FAM- HEX+, containing no *INS target DNA* but contains reference copies. Blue cluster: FAM+ HEX-, containing *INS target DNA* but no reference copies. Orange cluster: FAM+ HEX+ containing both *INS target DNA* and reference copies. The control samples are both split in two and either treated with or without methylation sensitive restriction enzyme (MSRE). The unmethylated *INS*-DNA fraction is calculated: $1-\frac{\frac{\left[INS\right]}{\begin{array}{c} \left[TTC5\right] \end{array}}\;with\;MSRE}{\frac{\begin{array}{c} \left[INS\right] \end{array}}{\left[TTC5\right]}\;without\;MSRE\;}*100\%$ . Abbreviations: ddPCR, Digital Droplet polymerase Chain Reaction; INS, Insulin; MSRE, Methylation Sensitive Restriction Enzyme.

First, the assay was validated in cell line models. DNA was isolated from EndoC-βH1 cells, a cell line that was derived from human β-cells(17). The MSRE duplex digital PCR assay was performed. This results in two-dimensional plots that demonstrate four different clusters each of them representing different DNA containing droplets (Figure 2). The green cluster contains no *INS target DNA* but only TTC5 copies; the blue cluster contains only *INS target DNA* but no TTC5 copies; the orange cluster contains both *INS target DNA* and TTC5 copies; the gray cluster includes the empty droplets. Without treatment of the MSRE (Figure 2A), the *INS target DNA* reflects the quantification of both unmethylated and methylated *INS target DNA*. After treatment with the MSRE HpaII (Figure 2B), the unmethylated *INS target DNA* is digested, resulting in less blue and orange droplets. For both, with and without treatment of MSRE, a stable independent reference, TTC5, was used to correct for input differences as it is not digested by the MSRE. When using both ratios from *INS target DNA* and reference TTC5 in the samples with and without treatment with MSRE, an unmethylated *INS*-DNA fraction of 98.1% (95% CI 97.3–98.8) was determined (Figure 2C). With regards to DNA isolated from THP-1 cells, both ratios from *INS target DNA* and reference TTC5, when treated with (Figure 2E) or without (Figure 2D) the MSRE HpaII, were calculated and this resulted in an unmethylated *INS*-DNA fraction of 3.5% (95% CI -5.2–11.5) (Figure 2F).

**FIGURE 3:**
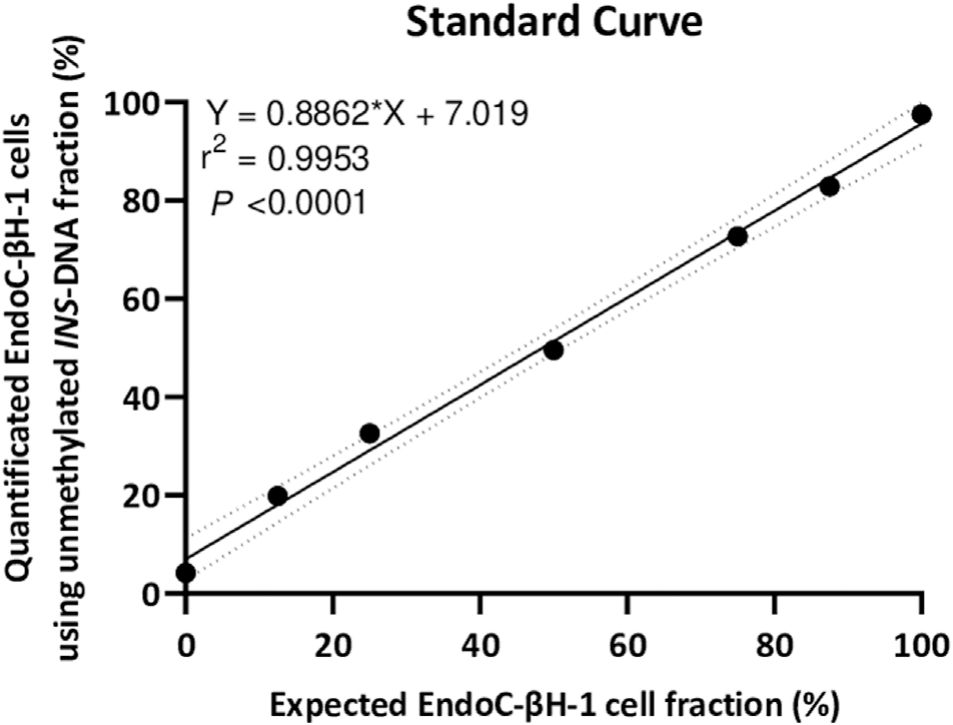
A seven point standard curve demonstrates the relation between input percentage of EndoC-βH-1 cells DNA (diluted in a background of THP-1 cells DNA) that could be expected and EndoC-βH-1 cells DNA quantified as unmethylated *INS*-DNA was measured using digital PCR. (Y = 0.8862 *X + 7.019, *r* ^2^ = 0.9953, *p* < 0.0001). Abbreviations: PCR; polymerase Chain Reaction.

As isolated DNA from EndoC-βH1 cells was essentially unmethylated for the *INS target DNA* whilst isolated DNA from THP-1 cells was mainly methylated for the *INS target DNA*, a 7-points standard curve was generated to technically validate the quantitative experimental setup. Isolated DNA from EndoC-βH1 cells diluted in the background of isolated DNA from THP-1 cells resulted in a strong linear correlation (*r* ^2^ = 0.9953, Y = 0.8862*X + 7.019, *p < 0.0001*) (Figure 3).

**FIGURE 4:**
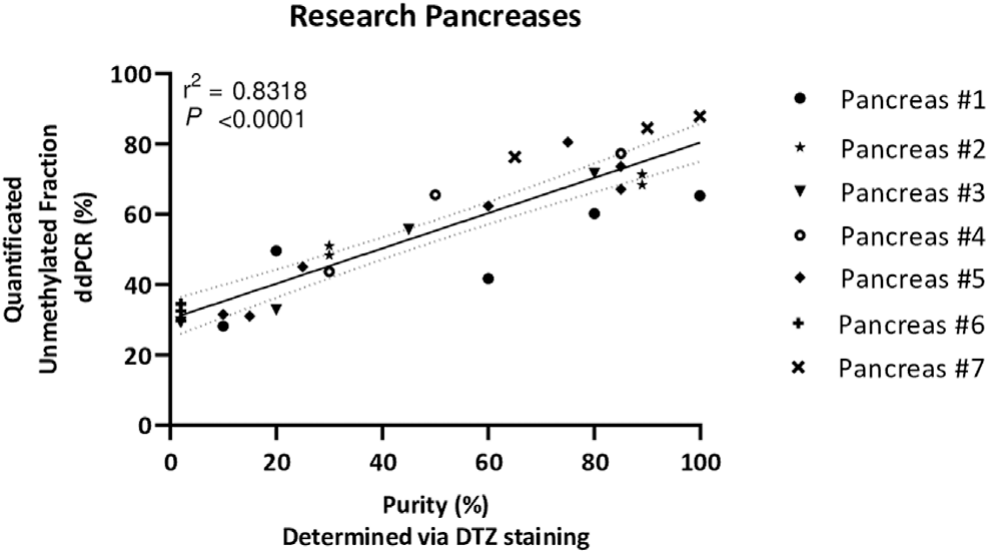
Unmethylated *INS*-DNA fraction quantified by digital PCR in different purities of islets, determined via DTZ staining, after isolation from seven donor research pancreases. (*r* ^2^ = 0.8318, *p* < 0.0001). Abbreviations: DTZ; Dithizone Staining, PCR; polymerase Chain Reaction.

Next, the unmethylated *INS*-DNA fraction was determined in 24 human islets preparations which were isolated from seven different human donor pancreases obtained for research. For each sample, islet purity was determined, varying from <5 to 99%, via dithizone staining which is currently used by most centers to estimate the fraction of pancreatic islets in an isolated islet preparation (18, 19). In the case of a sample with <5% purity, the sample was categorized as islet depleted tissue (i.e., pancreatic tissue left over from islet isolation). After using this MSRE duplex digital PCR assay on DNA isolated from all the different purities of the islets, the unmethylated *INS*-DNA fraction was quantified (Figure 4). When comparing the purity of the pancreatic islets a significant linear correlation was observed (R squared = 0.8318, *p < 0.0001*). In the samples containing islet depleted tissue an unmethylated *INS*-DNA fraction of 29.4%–34.5% was observed.

## Discussion

Previous studies have demonstrated that the human *INS* gene is controlled epigenetically by methylation as it is unmethylated in β-cells and methylated in most other cell types (4, 20-22). When cells are dying or lysed - either *in vivo* or for experimentation purposes - their genomic DNA is released into the milieu. This makes unmethylated-*INS* DNA a highly interesting marker to detect the death of β-cells. Several research groups have developed assays to measure the circulating fraction of unmethylated *INS*-DNA in humans, often aiming to be used in the context of early detection of β-cell death in type 1 Diabetes. In 2020 Speake et al. (23) assessed the performance of three different methodologies (5, 9 11) to quantify circulating levels of unmethylated *INS*-DNA in patients undergoing total pancreatectomy and subsequent islet auto-transplantation. This was considered a reliable model as damage or cell death of β-cells is known to occur during transplantation. Not only did the group measure a different CpG site or sites in the human *INS* gene in these three assays, they also applied different sample collection methods and measurement techniques (e.g., next generation sequencing or digital PCR). We agree with Speake’s group that to further develop these assays, optimization of the three different techniques might be beneficial. A similarity between all three assays was that DNA was treated with sodium bisulfite. This technique, which was first described by Frommer et al. (24), is still regarded as the gold standard to analyze DNA methylation. To prevent partial conversion and subsequent misinterpretation, the chemical conversion is performed at high concentrations. As a result, however, fragmentation and degradation of DNA will occur that may lead to an incorrect quantitative interpretation (13, 25). In addition, with regard to the bisulfite conversion kits used in these studies focusing on unmethylated-*INS* DNA, it remains a relatively time consuming technique e.g., as approximately 12–16 h are needed for the incubation period.

To circumvent or even avoid these limitations, we report in this protocol a proof-of-concept study where we have combined the MSRE with digital PCR techniques to measure unmethylated-*INS* DNA. As an MSRE can differentiate between methylated and unmethylated alleles, MSRE treatment for only 1 hour results in digestion of unmethylated DNA, with the methylated DNA remaining intact. This allows for the rapid calculation of the fraction of unmethylated alleles in our target of interest (*INS target DNA*). When using two different cell lines, a strong correlation was observed (Figure 3) demonstrating a high sensitivity and specificity of this assay.

Next, we extended the use of this assay to measure the unmethylated *INS*-DNA fraction in different purities of islets obtained after pancreas isolation (Figure 4). Interestingly, the purity of the samples was not directly proportional to the quantified unmethylated *INS*-DNA fraction as was found in the standard curve obtained from the 2 cell lines (Figure 3). When using the MSRE duplex digital PCR in islet depleted tissue (i.e. containing <5% islets) an unmethylated *INS*-DNA fraction of 29.4–34.5% was observed. Of note is that this observed fraction is likely not a limitation of the assay itself but an indication that the biological variability in methylation of the human *INS* gene promotor in non β-cells may play an important role. Our result is in line with the study by Kuroda et al. (22) who investigated nine CpG sequences in the human *INS* gene promotor and compared the methylation pattern in this region in the ‘islet cell fraction’ and in the ‘non-islet cell fraction’. In their study they demonstrated that the human *INS* gene promotor was mainly unmethylated in the islet cell fraction and predominantly methylated in the non-islet cell fraction (i.e., 13 of 15 clones (86%) in the non-islet cell fraction exhibited at least one unmethylated CpG out of the nine CpG sequences investigated).

With regard to the samples including high purity of islets, the quantified unmethylated *INS*-DNA fraction did not reach 100% which could be explained as the ratio of β-cells versus non-β-cells (e.g., alpha and delta) in human islets is generally assumed to be 50–70% (26). This is in line with the ±70% unmethylated *INS*-DNA fraction we have found (Figure 4).

A limitation of this proof-of-concept study is that our protocol was performed in cell lines and in different purities of human islet preparations obtained after isolation. Further validation experiments of this assay during islet isolation, islet culture and subsequent islet transplantation are necessary. During these next steps of the process an unknown amount of β-cell destruction occurs. To be able to specifically quantify the amount of β-cell loss using this promising assay could be helpful to differentiate between low or high quality and viability of islet preparations (12, 27). In clinical islet transplantation the accurate determination of the number of (viable) β-cells in a pancreatic islet preparation is essential. Not only assessment of the islet depleted tissue fraction, but more important the total number of isolated islets in the preparation is key for a successful transplant (28). In islet transplantation, the islet yield has previously been determined using various methods such as size-dependent islet counting by visualizing islets under a microscope and subsequent measurement of their volume (19), calculating both islet purity and graft volume or specific β-cell counting (28-31). To date, in most centers the estimation of the fraction of pancreatic islets in an isolated islet preparation is based on a method that uses dithizone staining (DTZ) (18, 19). Dithizone is a zinc chelating agent that, when added to an islet prep, results in a rapidly and reversibly red staining of islets which can therefore be distinguished from exocrine tissue. Importantly, this method cannot be used to determine the total number of β-cells in an isolated islet preparation. In addition, in case of β-cell degranulation, the red staining will not take place. Therefore, due to the human error that is intrinsic to this subjective method, an over- or under-estimation of the islet equivalent (IEQ) may easily occur. As such, determination of IEQ by eye or by digital image analysis has proven difficult within and between different centers (32).

Based on these notions, we suggest in this preliminary study that our newly developed MSRE duplex digital PCR assay using unmethylated *INS*-DNA may be a fast and easy method to specifically quantify β-cells. As shown previously, the combination of MSRE with digital PCR provides both specificity and sensitivity by quantitative assessment of target alleles (14). By measuring the concentration of the targeted unmethylated *INS*-DNA and therefore the number of lysed β-cells, this combined technique may be a promising tool to determine the fraction of β-cells immediately after islet isolation, during culture and immediately prior to islet transplantation. Pending further validation trials, the MSRE duplex digital PCR assay using unmethylated INS-DNA may therefore help decision making on islet quality (through the measurement of β-cell death) and islet quantity in islet transplantation centers.

## Data Availability

The raw data supporting the conclusions of this article will be made available by the authors, without undue reservation.

## Abbreviations

ddPCR, Digital Droplet polymerase Chain Reaction; DNA, DeoxyriboNucleic Acid; DTZ, Dithizone Staining; IEQ, Islet Equivalent; INS, Insulin; MSRE, Methylation Sensitive Restriction Enzyme; PCR polymerase Chain Reaction; T1D, Type 1 Diabetes; TTC5, Tetratricopeptide Repeat Domain 5.
