# Supplementary figures and images for: Quantification of Unmethylated Insulin DNA Using Methylation Sensitive Restriction Enzyme Digital Polymerase Chain Reaction
Source: Transpl Int. 2022 Apr 7;35:10167. doi: 10.3389/ti.2022.10167 (PMC9022224; doi:10.3389/ti.2022.10167)

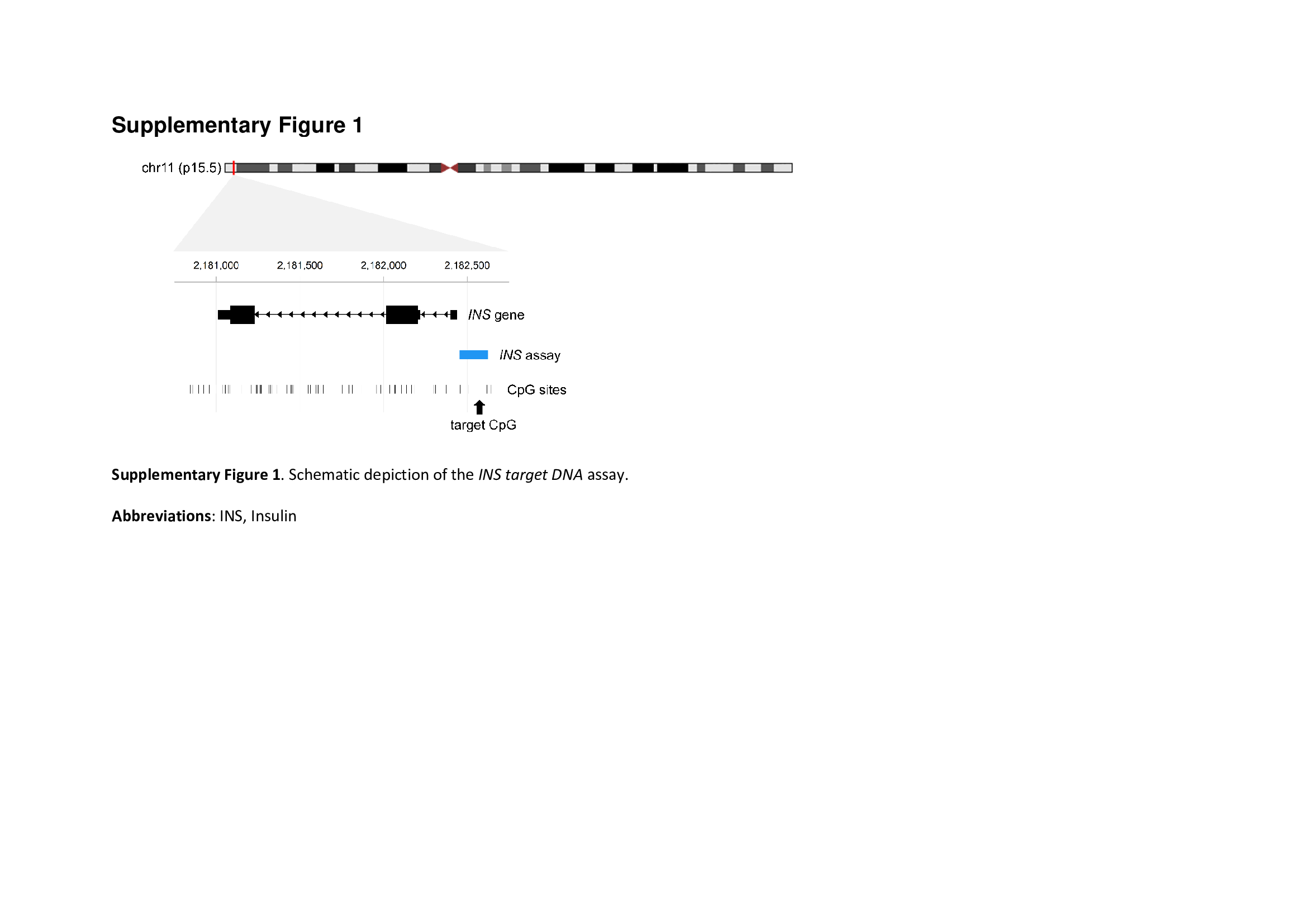

Supplement: Supplementary file 1 [file Image1.TIFF]
