# Supplementary material for: Quantification of Unmethylated Insulin DNA Using Methylation Sensitive Restriction Enzyme Digital Polymerase Chain Reaction
Source: Transpl Int. 2022 Apr 7;35:10167. doi: 10.3389/ti.2022.10167 (PMC9022224; doi:10.3389/ti.2022.10167)
Supplement: Supplementary file 2 [file Table1.DOCX]

| **Supplementary Table 1. Details primer, probe and methylation sensitive restriction enzyme information for digital polymerase chain reaction.** | |
| --- | --- |
| Assay *INS* chr. 11 (Sigma-Aldrich) | |
| Context Sequence | **GCTGACGACC AAGGAGATC**T *TCCCACAGAC CCAGCACCAG* GGAAATGGTC CGGAAATTGC AGCCTCAGCC CCCAGCCATC TGCCGACCCC CCCACCCCAG GCCCTAATGG GCCAGGCGGC AGGGGTTGAG AGGTAGGGGA GATGGGCT**CT GAGACTATAA AGCCAGCGGG** |
| Forward primer | 5’ **GCTGACGACCAAGGAGATC** 3’ |
| Reverse primer | 5’ **CCCGCTGGCTTTATAGTCTCAG** 3’ |
| Probe | 5’ *TCCCACAGACCCAGCACCAG* 3’ |
| Location | chr11: 2182455-2182624 |
| Amplicon length | 170 bp |
| Label | FAM |
| MSRE (HpaII) | Cut site  C \| C G G |
|  | |
| Assay *TTC5* reference chr. 14q (BioRad) | |
| Context Sequence | TGGTCGCGAT GCCACTGTGG CAACAGCCTG GCTGCTGGAT CCCTGAGGCT TCCCATTCAC CACTAGCAGG AGGGGCGTCT CCACTCGAAC ACTGGAAAAG GAATAGTCCT AGAAAAGACA GAC |
| Location (hg19) | chr14:20757798-20757920 |
| Amplicon length | 59 nucleotide |
| Label | HEX |
|  | |
