# Supplementary material for: Quantification of Unmethylated Insulin DNA Using Methylation Sensitive Restriction Enzyme Digital Polymerase Chain Reaction
Source: Transpl Int. 2022 Apr 7;35:10167. doi: 10.3389/ti.2022.10167 (PMC9022224; doi:10.3389/ti.2022.10167)
Supplement: Supplementary file 3 [file DataSheet1.DOCX]

**SUPPLEMENTARY MATERIAL**

**2 | METHOD**

Culture of human insulinoma EndoC-βH1 cells (Univercell- Biosolutions^15^, Toulouse, France), human monocytic leukaemia THP-1 cells and isolation and culture of human islets

*2.1 EndoC-βH1 cells*

Human insulinoma EndoC-βH1 cells were cultured in low glucose (1g/L) DMEM + Glutamax (Gibco, Breda, the Netherlands) supplemented with 2% (200g/L) Albumin (Sanquin Bloodbank, Leiden, the Netherlands), 10mM (300mg/mL) Nicotinamide (prepared by our institute’s pharmacy), 55µL Transferrin (100mg) and 6.7µL Selenite (0.5mg/mL) (both Sigma-Aldrich, Darmstadt, Germany).

*2.2 THP-1 cells*

Human monocytic THP-1 cells were cultured in RPMI 1640 (Gibco) with 2mM L-glutamine (Gibco) and 25 mM HEPES (Gibco) supplemented with 10% heat-inactivated fetal bovine serum (Biowest, Nuaillé, France) at 37 degree maintaining 5% CO2.

*2.3 Human islets*

NOTE: Human donor pancreases were used that could not be used for clinical purposes according to national criteria. Written informed consent from donors for research use of pancreatic tissue was obtained.

Human islets were isolated from seven donor pancreases in our institute according to the modified Ricordi method as previously described.^16, 17^

Volume and purity were determined by dithizone staining.^18, 19^ Islet depleted tissue contained <5% islets.

Human islets were cultured in CMRL 1066 medium (Corning-Mediatech, Herndon, VA, USA) supplemented with 2mg/mL Ciproxin (Bayer healthcare AG, Leverkusen, Germany), 50mg/mL gentamycin (Gibco), 200mM L-Glutamine (Gibco), 1M HEPES (Gibco), 300mg/mL Nicotinamide (prepared by our institute’s pharmacy), and Human Serum (Sanquin Bloodbank) at 37 degree for 1 to 4 days. Culture media was changed every two days.
